# Cell free DNA in patients with pancreatic adenocarcinoma: clinicopathologic correlations

**DOI:** 10.1038/s41598-024-65562-8

**Published:** 2024-07-08

**Authors:** Talent Theparee, Michael Akroush, Linda M. Sabatini, Vivien Wang, Kathy A. Mangold, Nora Joseph, Susan Jane Stocker, Alexa Freedman, Donald L. Helseth, Mark S. Talamonti, Karen L. Kaul

**Affiliations:** 1 Department of Pathology and Laboratory Medicine, NorthShore University Health System, Evanston, IL USA; 2 Department of Surgery, NorthShore University Health System, Evanston, IL USA; 3 Present Address: Department of Pathology, Chulalongkorn University Faculty of Medicine, Bangkok, Thailand; 4 Present Address: Department of Pathology, University of Michigan School of Medicine, Ann Arbor, MI USA; 5 Present Address: Northwestern University School of Medicine, Chicago, IL USA

**Keywords:** Cancer, Biomarkers, Molecular medicine

## Abstract

Detection of circulating tumor DNA (ctDNA) from plasma cell free DNA (cfDNA) has shown promise for diagnosis, therapeutic targeting, and prognosis. This study explores ctDNA detection by next generation sequencing (NGS) and associated clinicopathologic factors in patients with pancreatic adenocarcinoma (PDAC). Patients undergoing surgical exploration or resection of pancreatic lesions were enrolled with informed consent. Plasma samples (4–6 ml) were collected prior to surgery and cfDNA was recovered from 95 plasma samples. Adequate cfDNA for NGS (20 ng) was obtained from 81 patients. NGS was performed using the Oncomine Lung cfDNA assay on the Ion Torrent S5 sequencing platform. Twenty-five patients (30.9%) had detectable mutations in *KRAS* and/or *TP53* with allele frequencies ranging from 0.05 to 8.5%, while mutations in other genes were detected less frequently and always along with *KRAS* or *TP53*. Detectable ctDNA mutations were more frequent in patients with poorly differentiated tumors, and patients without detectable ctDNA mutations showed longer survival (medians of 10.5 months vs. 18 months, *p* = 0.019). The detection of circulating tumor DNA in pancreatic adenocarcinomas is correlated with worse survival outcomes.

## Introduction

Pancreatic adenocarcinoma (PDAC) is a leading cause of cancer deaths and is often detected in advanced stages when it is often inoperable^1^. The median overall survival in patients with pancreatic adenocarcinoma is approximately 7 months, with significantly better survival in patients with resectable tumors^2^. One of the only ways to provide an opportunity of long-term survival is surgical removal of the tumor with adjuvant chemotherapy. While surgical technique has advanced, many patients are still surgically unresectable or on the borderline of resectability^3^.

The most common genetic alterations in pancreatic ductal adenocarcinoma are mutations in *KRAS* and *TP53*, as well as less prevalent alterations in other driver genes such as *CDKN2A*, *SMAD4*, and in DNA damage repair genes^4^. *KRAS* mutations are present in up to 95% of pancreatic adenocarcinomas while TP53 alterations can be detected in up to 75%^5^.

Detection of circulating tumor DNA (ctDNA) from plasma cell free DNA (cfDNA) has shown promise for diagnosis, therapeutic targeting, and monitoring of pancreatic adenocarcinoma^6,7^. Multiple extraction methods and platforms including digital droplet PCR and next generation sequencing have been designed for detection of ctDNA with differing detection rates, input DNA requirements, and sensitivities^8–10^. These techniques have shown good concordance with matched tumor controls and a tendency to correlate with poorer overall survival^8,9,11,12^. The post-operative detection of ctDNA has also been associated with higher recurrence rates and worse outcomes^13^. ctDNA detection may also shed light on tumor evolution over time^7^.

This study explores clinicopathologic factors associated with ctDNA detection in patients with pancreatic ductal adenocarcinomas.

## Materials and methods

### Patient selection and plasma collection

This study received ethical approval from the Institutional Review Board of NorthShore University HealthSystem (Approval EH16-083). All methods were performed in accordance with the relevant guidelines and regulations. Patients who underwent surgical exploration or resection of pancreatic lesions from 2007 to 2014 were enrolled in this study with informed consent which was contained from the subjects, with samples collected into the institutional biorepository. Eight to twelve ml EDTA blood was collected from 3 h to 2 weeks prior to surgery. Most specimens were collected at the primary hospital and centrifuged (1200 g, 10 min) and the plasma stored at − 80 °C within 2 h until cell-free DNA isolation, though some samples were sent from other hospitals. All were processed within 24 h. Patients with pancreatic ductal adenocarcinoma were subsequently selected for inclusion. Pathological and clinical staging parameters and outcomes were obtained via the electronic medical record.

### Plasma cell free DNA recovery

cfDNA was recovered from 4 to 6 ml of plasma using a column purification method optimized to enrich for circulating cfDNA (Zymo Research Quick-cfDNA™ Serum & Plasma Kit, Irvine, CA) and further concentrated using the DNA Clean & Concentrator (Zymo Research). Cell free DNA recovery was assessed using the 4200 TapeStation (Agilent Technologies, Santa Clara, CA).

### FFPE DNA extraction

Selected cases with adequate tumor tissue and representing all tumor grades, stages, and survival times were collected. Tissues were selected from patients with and without identified ctDNA mutations. DNA was extracted using the Pinpoint Slide DNA Isolation System (Zymo Research).

### Next generation sequencing (NGS)

18.9–50.0 ng of input cfDNA was evaluated using the Oncomine™ Lung cfDNA Assay (ThermoFisher, Waltham, MA) on the Ion Torrent S5 sequencing platform. This is a limited, hotspot panel that was available at the time, in use in the lab, and optimized to enable sensitive detection of low level mutations in ctDNA and includes hotspots in *ALK*, *BRAF*, *EGFR,* ERBB2, *KRAS*, *MAP2K1*, *MET*, *NRAS*, *PIK3CA*, *ROS1*, and *TP53,* with interpretation focused on pathogenic variants in *KRAS* and *TP53*. Samples were sequenced to a mean depth of coverage of 17,589–113,777 × and a unique molecular coverage of 2276–11,382x. A 30–50 ng input with 4000 × molecular coverage provides a sensitivity down to 0.05% variant allele fraction. Additionally, 0.1% multiplex I cfDNA HD779 (Horizon Discovery, St Louis, MO) cfDNA reference standards were sequenced as a comparison.

Tissue DNA was sequenced using our in-house clinical Cancer HotSpot panel using the Ion AmpliSeq™ Cancer Hotspot Panel v2 (ThermoFisher) and an in-house bioinformatics pipeline^14^.

### Statistical analyses

Statistical analyses using the Chi-squared test, Fisher’s exact test, Mann–Whitney U test, and linear regression were utilized for correlation of clinical and pathologic parameters. A multivariable Cox proportional hazards model was used to test the association between mutation status and survival time. The model included adjustment for tumor stage, age, and sex. Analyses were conducted using RStudio version 2022.2.0.443 and a *p*-value of 0.05 was used to determine statistical significance.

## Results

**Figure 1:**
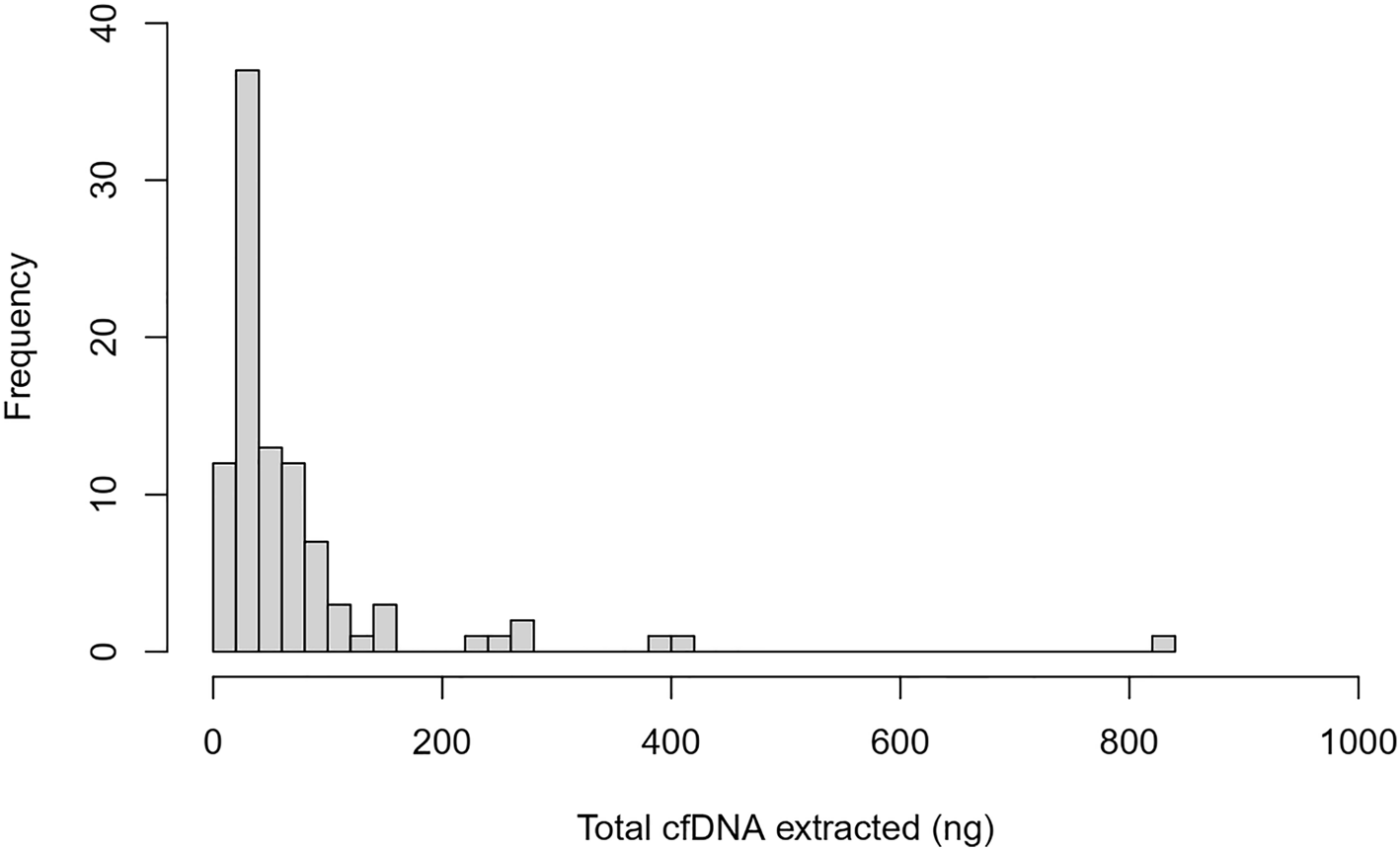
Total cfDNA extracted from patient specimens Total cfDNA extracted from patient plasma samples (ng).

Ninety-five patients were enrolled into this study and plasma specimens were obtained (Table 1). cfDNA was successfully extracted from EDTA preserved plasma and 81 samples yielded 18–50 ng of input DNA for NGS. A median of 37.3 ng of cfDNA was extracted from 4 to 6 ml of plasma, with a range of 6.8–836.8 ng (Fig. 1).

**Table 1:** Results of cfDNA extraction and ctDNA detection.

N	cfDNA recovery	cfDNA median (IQR)	cfDNA median concentration (IQR)	Mean depth of coverage of *KRAS*	cfDNA mutation in *KRAS* or *TP53*	*KRAS* mutations detected	*TP53* mutations detected	Allele frequencies detected
95	81 with adequate DNA	37.3 ng (26.6–75.8 ng)	7.0 ng/ml (4.6–12.6 ng/ml)	17,000–113,000x	25 (30.9%)	10 (12.3%)	18 (22.2%)	0.05–8.55%

By next generation sequencing, mutations in *KRAS* and *TP53* were detected in 25 of the 81 specimens (30.9%). *KRAS* mutations were detected in 10 (12.3%) and *TP53* mutations in 18 (22.2%) of cell-free DNA samples, including 3 samples with both *KRAS* and *TP53* mutations. Allele frequencies detected ranged from 0.05 to 8.55% (Table 1). Since samples negative for *KRAS* and *TP53* were also negative for other gene mutations, we only reported the 2 genes.

### Associations between assay parameters

There was no difference in the total cfDNA obtained from samples with detected or undetected circulating tumor DNA. There was no association between the amount of cfDNA obtained, ctDNA detection, and plasma volume (Table 2, S1 figure). The median depth of coverage was not significantly associated with total input cfDNA, but was associated with sequencing input and library ratios in pooling (S2 figure).

**Table 2:** Patient characteristics.

		Detected (n = 25)	Not detected (n = 56)	*p*-value
Age	Mean (SD)	68.8 (9.0)	66.1 (9.9)	0.242
Sex	F	7 (28.0)	24 (42.9)	0.306
	M	18 (72.0)	32 (57.1)	
Time_to_death, months	Median (IQR)	10.5 (5.5 to 23.0)	18.0 (10.0 to 36.0)	0.028
Stage	IA	1 (4.0)	3 (5.5)	0.111
	IB	4 (16.0)	2 (3.6)	
	IIA	4 (16.0)	4 (7.3)	
	IIB	8 (32.0)	32 (58.2)	
	IV	8 (32.0)	14 (25.5)	
Tumor_grade*	G1	4 (21.1)	19 (45.2)	< 0.001
	G2	4 (21.1)	19 (45.2)	
	G3	11 (57.9)	4 (9.5)	
Tumor size, mm**		40.2 (17.4)	28.8 (10.4)	0.028
Total_cfDNA_ng	Median (IQR)	42.5 (31.0 to 89.3)	46.2 (31.7 to 80.3)	0.842
cfDNA_conc_ng_ml	Median (IQR)	8.5 (5.6 to 14.9)	7.7 (5.3 to 13.4)	0.85
cfDNA_input_ng	Mean (SD)	36.8 (12.4)	38.0 (12.0)	0.676
Median_Depth of Coverage	Mean (SD)	50,703.2 (17,345.1)	49,590.3 (16,633.5)	0.784

*Tumor grade missing for 20.

**Tumor size missing for 50.

### Clinico-pathologic associations

**Figure 2:**
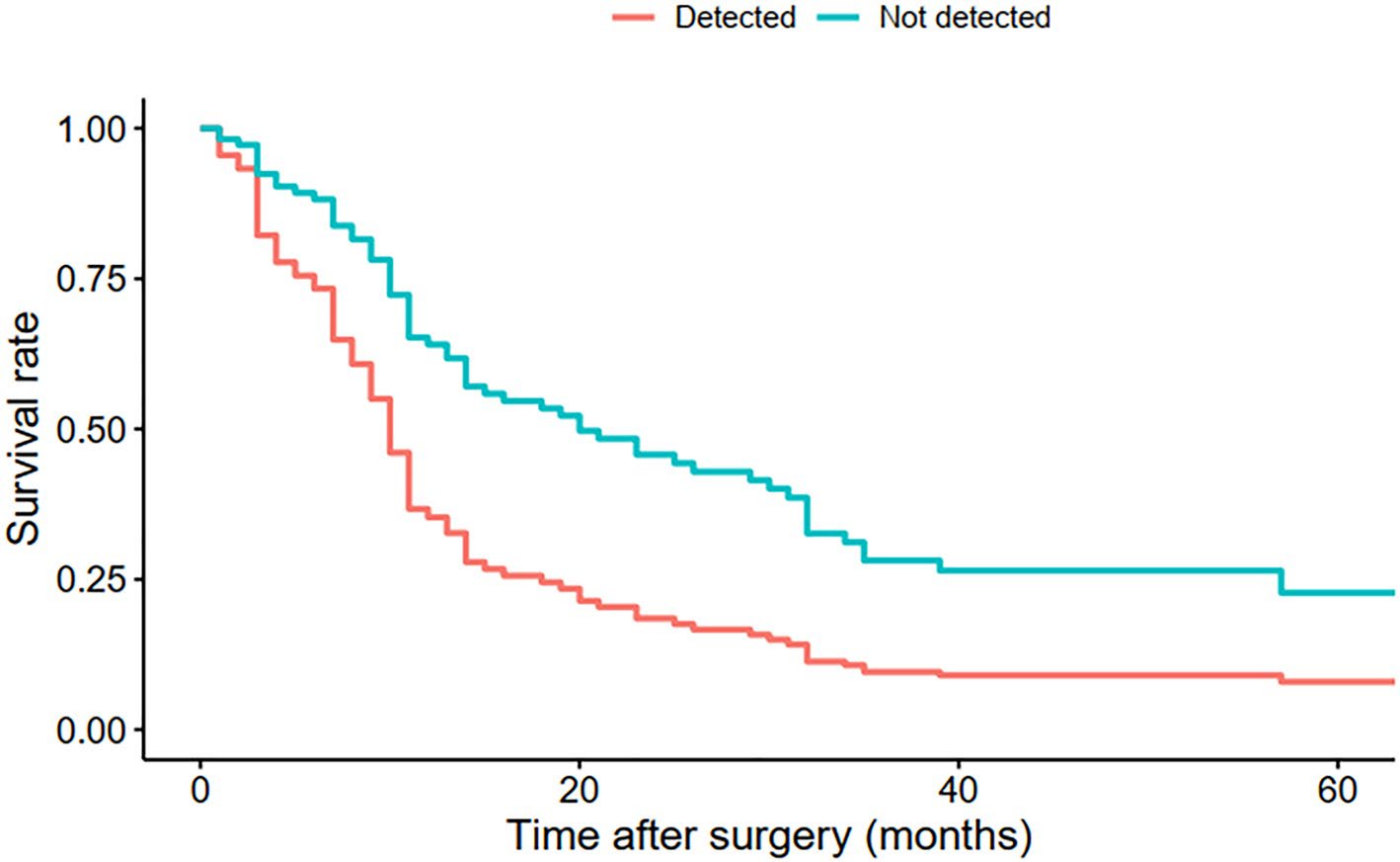
Adjusted survival curves from Cox regression.

Detectable ctDNA mutations in *KRAS* and *TP53* were more frequent in patients with poorly differentiated tumors (*p* < 0.001, Fig. 2). Tumor stage did not significantly influence detection of ctDNA mutations and no significant association was demonstrated between tumor stage and tumor grade (*p* = 0.90). Neoadjuvant status was not significantly associated with detection of ctDNA mutations (S2 Table).

The multivariable Cox model shows that detection of *KRAS* or *TP53* mutations in cfDNA were associated with survival outcome independent of tumor stage, age, and sex (medians of 10.5 vs 18 months, *p* = 0.019, Tables 1 and 2; Kaplan Meier curves presented in Fig. 2). Although tumor stage was associated with survival outcome (*p* < 0.001), it was not significantly associated with mutation detection.

### Association with tissue sequencing

DNA from 18 FFPE tissue specimens with adequate tissue were extracted and sequenced. *KRAS* alterations were detected in 17 of 18 cases and *TP53* alterations in 8 of 18 cases (S1 Table). In 12 cases of 18 cases showing either a *KRAS* or *TP53* mutation in the tissue specimen, there were no detectable mutations in cfDNA.

In six cases with detectable ctDNA mutations, four showed concurrent *KRAS* mutations in the tumor, and 2 cases showed *TP53* ctDNA mutation which was not present in the tumor. In 12 cases with no detectable mutations in cfDNA, all cases demonstrated either a *KRAS* or *TP53* mutation in the tissue specimen.

## Discussion

The detection rate of our ctDNA assay in this study is in line with previously published studies, including input cfDNA amounts and depth of coverage achieved. Multiple studies report a wide range of ctDNA mutation detection rates, from 12.5 to 88% using a single strand library preparation method^15–17^. The wide range can be partially attributed to variable methodologies (ddPCR, amplicon-based NGS, capture-based NGS, cell-free DNA extraction, plasma volume, input DNA amount) and patient cohorts (cancer stage, tumor volume)^18,19^. However, use for screening purposes will require improved sensitivity as even the combination of one method of ctDNA detection with four protein biomarkers resulted in a sensitivity of only 64%^20^. In this study, under 25% of the cohort had metastatic pancreatic adenocarcinoma, leading to a somewhat lower incidence of detectable mutations than expected in a study of metastatic patients.

The concordance between tissue and ctDNA mutations showed similar sensitivity with the entire cohort and confirmed the presence of identical *KRAS* alterations in all cases with *KRAS* alterations in ctDNA. On the other hand, two cases with detectable *TP53* in ctDNA did not have corresponding alterations in tumor tissue; these *TP53* mutations may be derived from other sources. Studies of clonal hematopoiesis have frequently detected *TP53* alterations detectable in cfDNA^21^.

The comparison of clinicopathologic parameters with ctDNA mutation status reveals a significant association of mutation status with both tumor grade and overall survival. Tumor necrosis has been associated with circulating tumor DNA and a correlation between tumor grade and necrosis was demonstrated in pancreatic ductal adenocarcinomas, which was also associated with patient survival^22^. Studies have also shown a relationship between tumor size, CA19-9 level, stage, and tumor volume on imaging with detection of ctDNA mutations^13,18,23^. The lack of a correlation between stage and detection of ctDNA mutations in our cohort may be due to the relatively low number of stage I patients and a larger number of stage IIB and stage IV cases in our patient population (S3 and S4 figure). However, stage was still an independent predictor of outcomes in our cohort even though it was not associated with ctDNA detection (Table 3).

**Table 3:** Cox multivariable regression model.

Variable	Regression co-efficient	Hazard ratio (95% confidence interval)	*p*-value
Mutation status (not detected)	− 0.702	0.49 (0.27–0.88)	0.017
Stage (non-IV)	− 0.682	0.50 (0.28–0.88)	0.018
Age	0.000	0.99 (0.97–1.02)	0.99
Sex (M)	1.251	1.25 (0.73–2.12)	0.40

The association of ctDNA mutation status with survival outcomes has been noted in a number of studies^8,9,12,17,24^, and post-operative detection of ctDNA mutations has also been reported as a predictor for recurrence^11,13^. Similar findings have been noted in other cancer types including colorectal^25^ and breast cancers^26,27^, highlighting the biological plausibility of ctDNA mutation status as an additional independent predictor of patient outcomes. This could represent a valuable tool for treatment decisions, particularly for malignancies such as PDAC which require surgical interventions that carry a high morbidity risk. However, prospective validation of this tool is required.

This study utilized banked specimens collected from 2007 to 2014. Our understanding of the impact of preanalytic parameters in the collection, processing, and storage on the recovery of ctDNA has greatly evolved in recent years^28,29^ However…add here.

Additionally, this study only compared the retrospective evaluation of patient clinico-pathologic data and pre-surgical plasma collection at a single time point. Other investigators have detected ctDNA prior to recurrence or progression using serial ctDNA measurements^13,30^.

## Conclusions

Detection of ctDNA mutations in PDAC is a promising test which requires further prospective validation as a predictive and prognostic tool to assist in patient care decisions.

### Supplementary Information

Supplementary Information.

## Data Availability

The datasets generated during and/or analyzed during the current study are not publicly available due to institutional data policies but are available from the corresponding author on reasonable request. Sequencing data has been deposited the NCBI repository (http://www.ncbi.nlm.nih.gov/bioproject/1046660).
