# Supplementary material for: Cell free DNA in patients with pancreatic adenocarcinoma: clinicopathologic correlations
Source: Sci Rep. 2024 Jul 8;14:15744. doi: 10.1038/s41598-024-65562-8 (PMC11231234; doi:10.1038/s41598-024-65562-8)
Supplement: Supplementary file 1 — Supplementary Information. [file 41598_2024_65562_MOESM1_ESM.docx]

**Supporting information**

**S1 figure: Plasma volume does not correlate with cfDNA concentration**


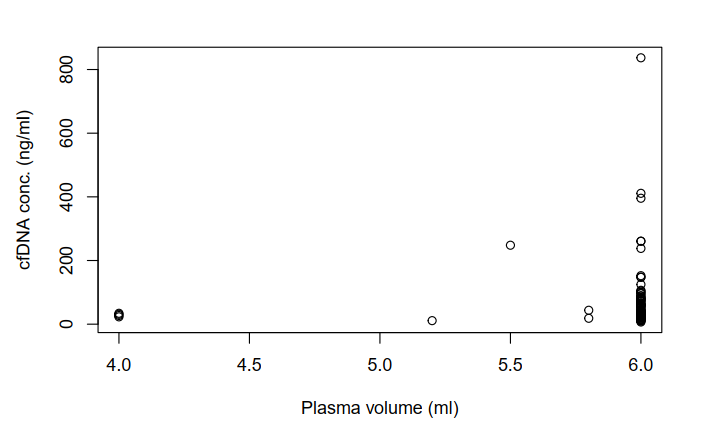


**S2 figure: The median depth of coverage is not correlated with input cfDNA**


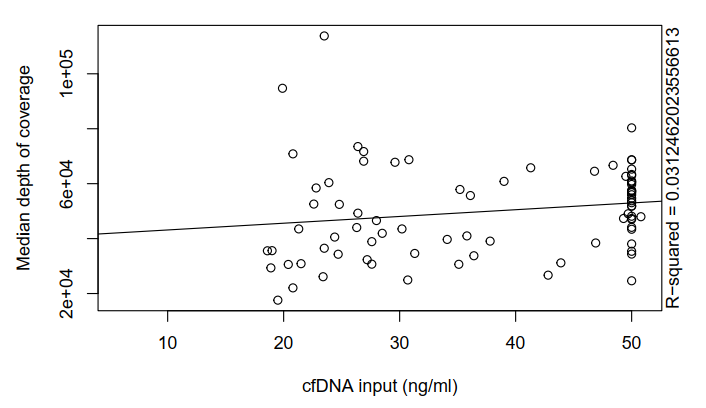


**S3 Figure: Tumor staging**


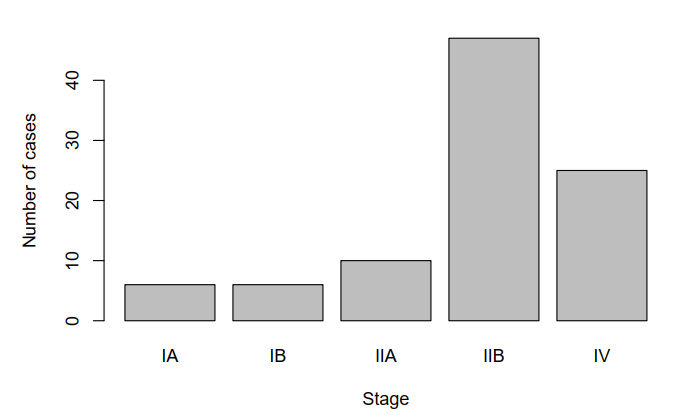


**S4 Figure: Relationship of overall cfDNA to tumor stage**


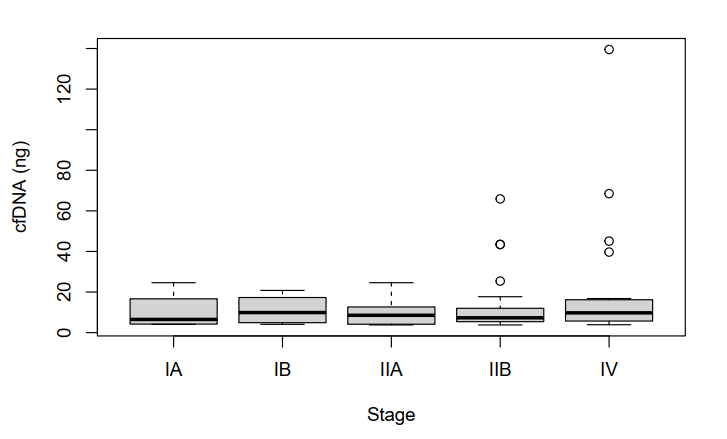


**S1 Table: Tissue and ctDNA concordance**

| **Case** | **Tumor *KRAS*** | **Tumor *TP53*** | **ctDNA *KRAS*** | **ctDNA *TP53*** |
| --- | --- | --- | --- | --- |
| 1293 | *KRAS* G12V, V8L | *TP53* G244S | *KRAS* G12V | Not detected |
| 1157 | *KRAS* G12V | Not detected | *KRAS* G12V | Not detected |
| 1082 | *KRAS* G12R | Not detected | *KRAS* G12R | Not detected |
| 1185 | *KRAS* G12D | Not detected | *KRAS* G12D | Not detected |
| 1210 | Not detected | Not detected | Not detected | *TP53* Y220C |
| 1033 | *KRAS* G12D | Not detected | Not detected | *TP53* G245C |
| 1104 | *KRAS* G12D | *TP53* L264fs | Not detected | Not detected |
| 0782 | *KRAS* G12D | Not detected | Not detected | Not detected |
| 1229 | *KRAS* G12R | *TP53* R175H | Not detected | Not detected |
| 0718 | *KRAS* G12D | Not detected | Not detected | Not detected |
| 0810 | *KRAS* G12D | *TP53* I195T | Not detected | Not detected |
| 1304 | *KRAS* G12D | *TP53* R175H | Not detected | Not detected |
| 1239 | *KRAS* G12D | Not detected | Not detected | Not detected |
| 1121 | *KRAS* G12D | Not detected | Not detected | Not detected |
| 1259 | *KRAS* G12V | *TP53* R248W | Not detected | Not detected |
| 1286 | *KRAS* G12D | *TP53* del29 E285fs | Not detected | Not detected |
| 1294 | *KRAS* Q61H | *TP53* V157F | Not detected | Not detected |
| 1065 | *KRAS* G12R | Not detected | Not detected | Not detected |

**S2 Table: Neo-adjuvant status and ctDNA detection**

|  | **Neo-adjuvant** | **No neo-adjuvant** | **Unknown** |
| --- | --- | --- | --- |
| **ctDNA mutation detected** | 6 | 19 | 0 |
| **Mutation not detected** | 23 | 46 | 2 |
| **Total** | 29 | 65 | 2 |
